# Supplementary material for: Electronic excitations and spin interactions in chromium trihalides from embedded many-body wavefunctions
Source: NPJ 2D Mater Appl. 2024 Aug 30;8(1):56. doi: 10.1038/s41699-024-00494-5 (PMC11364507; doi:10.1038/s41699-024-00494-5)
Supplement: Supplementary file 1 — Supplemental Material for the manuscript [file 41699_2024_494_MOESM1_ESM.pdf]

Supplementary Information

Electronic excitations and spin interactions in chromium trihalides from embedded many-body wavefunctions

Ravi Yadav,[†,‡] Lei Xu,[¶] Michele Pizzochero,[§] Jeroen van den Brink,[¶] Mikhail I. Katsnelson,[⊥] and Oleg V. Yazyev[*,†,‡]

†*Institute of Physics, Ecole Polytechnique Fédérale de Lausanne (EPFL), CH-1015 Lausanne, Switzerland*

‡*National Centre for Computational Design and Discovery of Novel Materials (MARVEL), Ecole Polytechnique Fédérale de Lausanne (EPFL), CH-1015 Lausanne, Switzerland*

¶*Institute for Theoretical Solid State Physics, IFW Dresden, Helmholtzstr. 20, 01069 Dresden, Germany*

§*School of Engineering and Applied Sciences, Harvard University, Cambridge, MA 02138, United States*

‖*Institute for Theoretical Physics and Würzburg-Dresden Cluster of Excellence ct.qmat, Technische Universität Dresden, 01069 Dresden, Germany*

⊥*Institute for Molecules and Materials, Radboud University, 6525AJ Nijmegen, The Netherlands*

E-mail: oleg.yazyev@epfl.ch

Supplementary Note 1: One-site quantum chemistry calculations

Multiplet structures (Table 1 in the main text) and intra-site magnetic interactions (Table 2 in the main text) are obtained using the finite-size model shown in Figure 1(b) in the main text. This model consists of a central unit that comprises a single CrX_6 (X=Cl, Br, I) octahedron treated with many-body wavefunctions, surrounded by the three nearest-neighbor octahedra. These latter octahedra account for the finite charge distribution in the vicinity of the central unit and are treated at the Hartree-Fock level. The remaining crystalline environment is modeled by arrays of point charges reproducing the ionic Madelung potential in the cluster region.[1] All-electron basis functions of quadruple-zeta quality supplemented with f-polarization functions are used for the Cr^{3+} ion in the reference unit.[2] The Cr^{3+} ions centered in the three nearest-neighbor octahedra are described as closed-shell Sc^{3+} ions with an all-electron basis set of triple-zeta quality.[2] For Cl and Br ligands in $CrCl_3$ and $CrBr_3$, respectively, an all-electron basis set of triple-zeta quality is employed.[3] The Cl and Br atoms in the octahedra surrounding the reference unit are represented with an all-electron basis sets of double-zeta quality.[3] In the case of CrI_3, energy-consistent relativistic pseudopotentials along with quadruple-zeta quality basis sets for the valence shells of I atoms in the reference octahedron,[4] while energy-consistent relativistic pseudopotentials along with triple-zeta quality basis sets are used for the I atoms in the nearest-neighbor octahedra.[4]

CASSCF wavefunctions are variationally optimized for an average of low-lying seven quartet and five doublet states. In the subsequent MRCI calculations, we account for dynamic correlation effects by including single- and double-excitations involving the t_{2g} orbitals of the Cr^{3+} ions and the p valence shells of halogen ligands within the central unit.[5] Calculations are performed using the MOLPRO package.[6]

Supplementary Note 2: Quantum chemistry simulation of XAS and RIXS spectra

The simulation of XAS and RIXS spectra (Figure 2 in the main text) is carried out at the CASSCF level using the finite-size model shown in Supplementary Figure 1(a).[7] This model consists of a single CrX_6 (X = Cl, I) octahedron surrounded by an array of point charges to reproduce the ionic Madelung potential of the crystalline environment.[1] For the Cr^{3+} ion, we use an all-electron triple-zeta Douglas-Kroll basis sets with diffuse functions, along with weighted core-valence sets aug-cc-pwCVTZ-DK[2] to describe core-valence correlation effects. For the halogen ligands, we employ an all-electron triple-zeta Douglas-Kroll basis sets with diffuse functions aug-cc-pVTZ-DK.[3] To compute the Cr^{3+} $3d^3$ valence-excited states at the CASSCF level, we consider an active space comprising five $3d$ orbitals (t_{2g} and e_g) and three electrons. Ten spin-quartet and forty spin-doublet valence states associated with this manifold are obtained in the state-averaged complete active space self-consist field optimization. The Pipek-Mezey localization scheme,[8] as implemented in Ref. 6, is adopted for localizing the orbitals of the halogen atoms. All these valence-excited states enter the spin-orbit coupling calculations at the CASSCF level. Spin-orbit coupling (SOC) effects are accounted for by diagonalizing the Breit-Pauli spin-orbit matrix in the basis of the scalar relativistic (SR) states.[9] Calculations are performed with the MOLPRO package.[6] The source code is modified and compiled in order to compute up to 800 SR states in the CASSCF module, thus allowing these states to be coupled in the subsequent SOC calculations. The resulting d excitation energies up to 4 eV are listed in Supplementary Tables 2 and 3.

For the determination of the $2p^5 3d^4$ core-hole states, which are the final states in the L-edge X-ray absorption process and the intermediate states in the L-edge RIXS process, the active space is defined in terms of nine electrons and five Cr $3d$ orbitals, in addition to three Cr $2p$ orbitals. The self-consistent field optimization is performed for an average of 15 spin sextet, 160 spin quartet, and 325 spin doublet states associated with the $2p^6 3d^3$ and $2p^5 3d^4$ configurations (the first 10 quartet and 40 doublet states in lower energies correspond to the

$2p^6 3d^3$ configurations). On top of this CASSCF reference, in the subsequent configuration interaction treatment, the occupation restriction of Cr $2p$ orbitals is defined as five electrons in order to exclude the $2p^6 3d^3$ valence-excited states, such that the corresponding CASSCF wavefunction including only 15 spin sextet, 150 spin quartet and 285 spin doublet states associated with the $2p^5 3d^4$ configurations. All these core-hole states further enter the SOC calculations, leading to a total of 1260 spin-orbit-coupled states.

The dipole transition matrix elements between wavefunctions expressed in terms of the nonorthogonal orbitals for the $3d^3$ and $2p^5 3d^4$ groups of states are derived according to the procedure described in Ref. 10. The XAS and RIXS scattering geometry and the directions of polarization are adjusted according to the setup of experimental measurements described in Ref. 11, also shown in Supplementary Figure 1(b). Linearly polarized x-rays are incident at an angle θ with the plane of the sample, this latter being oriented in such a way that the c-axis points to the surface normal. The scattering angle α between the incoming and outgoing light beams is set to 40° and the incident angle θ to 50°. The incoming light is linearly polarized, either perpendicularly to the scattering plane (σ polarization) or within the scattering plane (π polarization). For the outgoing radiation, we carry out a summation over the two independent polarization directions.

For the analysis of the *ab initio* wavefunctions, we rely on a local coordinate frame with the z component pointing along c axes of the CrX_3 unit cell. The rotation of the σ, π and π' (introduced as outgoing π polarization) vectors as function of the angle θ is described by the following geometrical relations

$$\begin{aligned}
\overrightarrow{D}_\sigma &= \overrightarrow{D}_y, \\
\overrightarrow{D}_\pi &= \overrightarrow{D}_x \sin\theta + \overrightarrow{D}_z \cos\theta, \\
\overrightarrow{D}_{\pi'} &= \overrightarrow{D}_x \sin(\theta+\alpha) + \overrightarrow{D}_z \cos(\theta+\alpha),
\end{aligned} \tag{1}$$

where $\overrightarrow{D}_{x,y,z} = e \cdot \overrightarrow{R}_{x,y,z}$ are the dipole transition matrix elements. The expression of the

X-ray absorption cross section[12,13] involves the summation over the ground state and core-excited states

$$I^{XAS}(\hbar\omega,\epsilon,\theta) = 4\pi^2\alpha\hbar\omega \sum_j \frac{1}{\mathrm{g_{gs}}} \sum_l \left| \left\langle \Psi^l_{\mathrm{c}^\star} \right| \overrightarrow{D}_\epsilon \left| \Psi^j_{\mathrm{gs}} \right\rangle \right|^2 \\ \times \frac{\Gamma_{\mathrm{c}^\star}/2\pi}{(E^j_{\mathrm{gs}} + \hbar\omega - E^l_{\mathrm{c}^\star})^2 + (\Gamma_{\mathrm{c}^\star})^2/4}. \quad (2)$$

The RIXS double differential cross-section is obtained by summing over the outgoing polarization directions[12,14]

$$I^{RIXS}(\hbar\omega,\hbar\omega',\epsilon,\theta) = \frac{\mathrm{d}^2\sigma^{\mathrm{RIXS}}_{\overrightarrow{k},\epsilon}}{\mathrm{d}\Omega'\mathrm{d}\hbar\omega'} = \frac{\alpha^2\hbar^2}{e^4c^2}\omega\omega'^3 \sum_{\epsilon'} \sum_j \frac{1}{\mathrm{g_{gs}}} \sum_k \left| \sum_l \frac{\left\langle \Psi^k_{\mathrm{fs}} \right| \overrightarrow{D}_{\epsilon'} \left| \Psi^l_{\mathrm{c}^\star} \right\rangle \left\langle \Psi^l_{\mathrm{c}^\star} \right| \overrightarrow{D}_\epsilon \left| \Psi^j_{\mathrm{gs}} \right\rangle}{E^j_{\mathrm{gs}} + \hbar\omega - E^l_{\mathrm{c}^\star} + (i\Gamma_{\mathrm{c}^\star}/2)} \right|^2 \\ \times \frac{\Gamma_{\mathrm{fs}}/2\pi}{(E^j_{\mathrm{gs}} + \hbar\omega - E^k_{\mathrm{fs}} - \hbar\omega')^2 + (\Gamma_{\mathrm{fs}})^2/4}. \quad (3)$$

In Equations 2 and 3, α is the fine structure constant, $\hbar\omega$ is the energy of the incoming photons, $\hbar\omega'$ is the energy of outgoing photons, while ϵ and ϵ' are polarizations of the incoming and outgoing photons, respectively. For I^{XAS}, the summations take into account all core-hole (intermediate) states and the possible degeneracy of the ground state $\mathrm{g_{gs}}$. For I^{RIXS}, the summations take into account all core-hole $2p^5 3d^4$ (intermediate) states and $2p^6 3d^3$ (final) states and the possible degeneracy of the ground state, $\mathrm{g_{gs}}$. The lifetimes of the core-excited and valence-excited states are $\Gamma_{\mathrm{c}^\star}$ and Γ_{fs}, respectively. The natural widths $\Gamma_{\mathrm{c}^\star}$ are set to 0.4 eV for both $CrCl_3$ and CrI_3. The natural widths Γ_{fs} are set to 0.3 eV for $CrCl_3$ and 0.35 eV for CrI_3, that is, the same values adopted in the reference experimental work of Ref. 11. The RIXS spectra of π-polarization direction are directly compared to the corresponding experimental spectra.[11]

Supplementary Note 3: Two-site quantum chemistry calculations

Inter-site magnetic interactions (Table 3 in the main text) are obtained using the finite-size model shown in Figure 1(c) in the main text. This model consists of a central unit that comprises two edge-sharing Cr_2X_{10} (X = Cl, Br, I) octahedra treated with many-body wavefunctions, surrounded by the four nearest-neighbor octahedra. These latter octahedra account for the finite charge distribution in the vicinity of the central unit and are treated at the Hartree-Fock level. The remaining crystalline environment is modeled by arrays of point charges reproducing the ionic Madelung potential in the cluster region.[1] All-electron basis functions of quadruple-zeta quality were used for the Cr^{3+} ions[2] in the two-octahedra central unit. The bridging Cl and Br ligands are modeled with an all-electron quintuple-zeta quality basis set,[3,15] while, for the bridging I ligand, energy-consistent relativistic pseudopotentials along with quintuple-zeta quality basis sets for the valence shells are employed.[4] The remaining ligand atoms in the central region are described using a triple-zeta quality basis set in the case of Cl and Br atoms,[3,15] and energy-consistent relativistic pseudopotentials along with triple-zeta quality basis sets in the case of I atoms.[4] Cr^{3+} ions centered at the octahedra adjacent to the reference unit are described as closed-shell Sc^{3+} ions and an all-electron triple-zeta basis functions.[2] The ligands belonging to these octahedra are modeled with double-zeta quality basis set for the Cl and Br atoms[3,15] and energy-consistent relativistic pseudopotentials along with double-zeta quality basis sets for the I atoms.[4]

CASSCF wavefunctions are variationally optimized for an average of one septet, quintet, triplet and singlet states, which are mainly of $t_{2g}^3 - t_{2g}^3$ character. In addition to $t_{2g}^3 - t_{2g}^3$ direct exchange between the nearest-neighbor sites, these wavefunctions consist of a finite-weight contribution that stems from inter-site excitations of the $t_{2g}^4 - t_{2g}^2$ type.[5] MRCI calculations account for single- and double-excitations involving the t_{2g} orbitals of the Cr^{3+} ions and the p valence shells of the bridging halogen ligands. In the treatment of spin-orbit effects, one septet, quintet, triplet and singlet states are considered, both in CASSCF and MRCI approaches. Calculations are performed using the MOLPRO package.[6]

Supplementary Table 1: Effect of in-plane compressive and tensile lattice strain (ϵ) on the multiplet structure of $CrCl_3$, as obtained at the MRCI level of theory, using the finite-size model shown in Figure 1(b) in the main text. Energies are given in eV and referenced to the ground state.

	$\epsilon = -2\%$	$\epsilon = 0\%$	$\epsilon = +2\%$
$^4A_2$ ($t_{2g}^3 e_g^0$)	0.00	0.00	0.00
$^4T_2$ ($t_{2g}^2 e_g^1$)	1.79, 1.79, 1.85	1.67, 1.68, 1.70	1.38, 1.45, 1.50
^2E ($t_{2g}^3 e_g^0$)	2.39, 2.40	2.21, 2.21	2.36, 2.37
$^4T_1$ ($t_{2g}^2 e_g^1$)	2. 78, 2.80, 2.84	2.50, 2.52, 2.59	2.25, 2.35, 2.41
$^2T_1$ ($t_{2g}^3 e_g^0$)	2.49, 2.51, 2.52	2.31, 2.33, 2.34	2.48, 2.49, 2.51
$^2T_1$ ($t_{2g}^2 e_g^1$)	3.35, 3.36, 3.38	3.03, 3.05, 3.07	3.23, 3.26, 3.28
$^2A_1$ ($t_{2g}^2 e_g^1$)	3.72	3.47	3.42
$^2T_1$ ($t_{2g}^2 e_g^1$)	3.99, 4.01, 4.04	3.76, 3.77, 3.78	3.67, 3.70, 3.75
$^4T_1$ ($t_{2g}^1 e_g^2$)	4.43, 4.44, 4.48	4.05, 4.07, 4.08	3.88, 3.93, 3.97

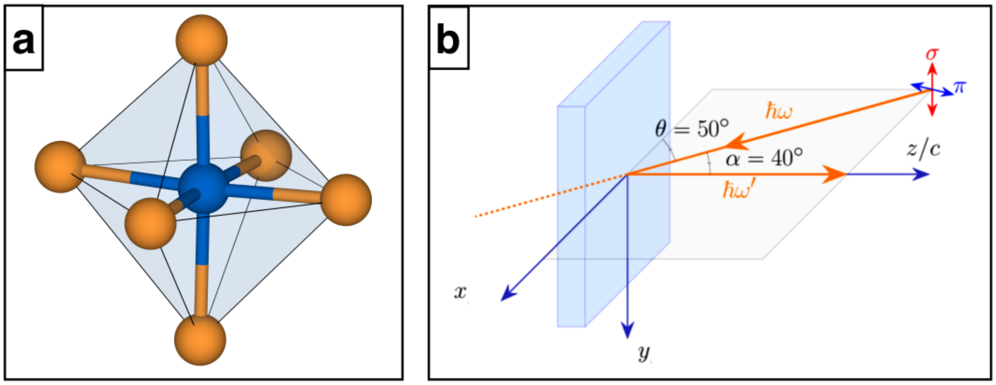

Supplementary Figure 1: (a) Embedding model used in the simulation of the XAS and RIXS spectra. Blue and orange spheres represent chromium and halogen atoms, respectively. The model is embedded in an array of points charges (not shown) to ensure charge neutrality and reproduce the crystalline environment. (b) RIXS scattering geometry used in our *ab initio* calculations, analogous to the experimental setup adopted in Ref. 11.

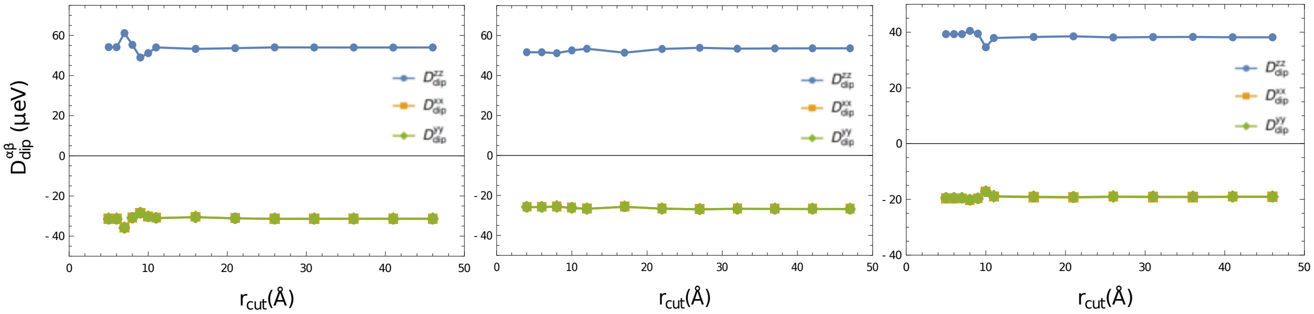

Supplementary Figure 2: Convergence of the diagonal elements of the dipolar anisotropy tensor $D^{\alpha\alpha}_{\rm dip}$ per transition metal ion for (a) $CrCl_3$, (b) $CrBr_3$ and (c) CrI_3 as a function of cutoff distance $r_{\rm cut}$.

Supplementary Table 2: Relative energies (in eV) at the CASSCF and CASSCF+SOC level of theory for the Cr^{3+} $3d^3$ multiplet structure in $CrCl_3$, as obtained using the finite-size model shown in Supplementary Figure 1(a). Each CASSCF+SOC value denotes a spin-orbit doublet. For the ^4T and ^2T states, only the lowest and highest components are given.

	Relative energy (eV)	
	CASSCF	CASSCF+SOC
$^4A_2(t_{2g}^3)$	0.00	0.00
$^4T_2(t_{2g}^2e_g^1)$	1.62; 1.66; 1.66	1.61 ... 1.67
$^2E(t_{2g}^3)$	2.27; 2.27	2.27; 2.28
$^2T_1(t_{2g}^3)$	2.36; 2.41; 2.41	2.36 ... 2.42
$^4T_1(t_{2g}^2e_g^1)$	2.56; 2.56; 2.67	2.55 ... 2.67
$^2T_2(t_{2g}^3)$	3.19; 3.19; 3.29	3.18 ... 3.30
$^2A(t_{2g}^2e_g^1)$	3.53	3.54
$^2T_1(t_{2g}^2e_g^1)$	3.75; 3.83; 3.83	3.75 ... 3.84
$^2T_2(t_{2g}^2e_g^1)$	3.95; 3.95; 4.01	3.93 ... 4.00

Supplementary Table 3: Relative energies (in eV) at the CASSCF and CASSCF+SOC level of theory for the Cr^{3+} $3d^3$ multiplet structure in CrI_3, as obtained using the finite-size model shown in Supplementary Figure 1(a). Each CASSCF+SOC value denotes a spin-orbit doublet. For the ^4T and ^2T states, only the lowest and highest components are given.

	Relative energy (eV)	
	CASSCF	CASSCF+SOC
$^4A_2(t_{2g}^3)$	0.00	0.00
$^4T_2(t_{2g}^2e_g^1)$	1.37; 1.41; 1.41	1.36 ... 1.41
$^2E(t_{2g}^3)$	2.19; 2.19	2.19; 2.19
$^4T_1(t_{2g}^2e_g^1)$	2.22; 2.22; 2.29	2.22 ... 2.30
$^2T_1(t_{2g}^3)$	2.29; 2.33; 2.33	2.30 ... 2.33
$^2T_2(t_{2g}^3)$	2.99; 2.99; 3.09	2.99 ... 3.09
$^2A(t_{2g}^2e_g^1)$	3.22	3.22
$^2T_1(t_{2g}^2e_g^1)$	3.37; 3.45; 3.45	3.37 ... 3.45
$^2T_2(t_{2g}^2e_g^1)$	3.59; 3.59; 3.63	3.58 ...
$^4T_1(t_{2g}^1e_g^2)$	3.59; 3.79; 3.79	... 3.79
$^2E(t_{2g}^2e_g^1)$	3.91; 3.91	3.90; 3.92

Supplementary Table 4: $\bar{\bar{D}}_{\text{sia}}$ tensor as obtained from MRCI calculations in the units of cm^{-1} for CrX_3 (X = Cr, Br, I).

$CrCl_3$	$CrBr_3$	CrI_3
$\begin{bmatrix} 0.176 & -0.006 & -0.012 \\ -0.006 & 0.194 & 0.009 \\ -0.012 & 0.009 & -0.123 \end{bmatrix}$	$\begin{bmatrix} 0.360 & -0.003 & 0.002 \\ -0.003 & 0.442 & 0.012 \\ 0.002 & 0.012 & -0.304 \end{bmatrix}$	$\begin{bmatrix} 0.642 & -0.018 & -0.009 \\ -0.018 & 0.575 & 0.011 \\ -0.009 & 0.011 & -0.3596 \end{bmatrix}$

Supplementary Table 5: Heisenberg exchange parameter (in meV) in CrX_3 (X = Cr, Br, I) obtained by considering the isotropic bilinear Heisenberg Hamiltonian (i.e., neglecting the biquadratic and inter-site anisotropic terms in Equation 3 of the main text.

	$CrCl_3$		$CrBr_3$		CrI_3	
	CASSCF	MRCI	CASSCF	MRCI	CASSCF	MRCI
J_1 (meV)	−0.69	−1.08	−0.67	−1.28	−0.66	−1.42

Supplementary References

(1) Klintenberg, M.; Derenzo, S.; Weber, M. Accurate crystal fields for embedded cluster calculations. *Computer Physics Communications* **2000**, *131*, 120.

(2) Balabanov, N. B.; Peterson, K. A. Systematically convergent basis sets for transition metals. I. All-electron correlation consistent basis sets for the 3d elements Sc-Zn. *The Journal of Chemical Physics* **2005**, *123*, 064107.

(3) Woon, D. E.; Dunning, T. H. Gaussian basis sets for use in correlated molecular calculations. III. The atoms aluminum through argon. *The Journal of Chemical Physics* **1993**, *98*, 1358.

(4) Peterson, K. A.; Shepler, B. C.; Figgen, D.; Stoll, H. On the spectroscopic and thermochemical properties of ClO, BrO, IO, and their anions. *The Journal of Physical Chemistry A* **2006**, *110*, 13877.

(5) Helgaker, T.; Jørgensen, P.; Olsen, J. *Molecular Electronic-Structure Theory*; Wiley, Chichester, 2000.

(6) Werner, H. J.; Knowles, P. J.; Knizia, G.; Manby, F. R.; Schütz, M. Molpro: A general-purpose quantum chemistry program package. *WIREs Computational Molecular Science* **2012**, *2*, 242.

(7) Xu, L. *Ab initio modeling of the electronic structure of d-metal systems and of resonant inelastic x-ray scattering responses*; Ph.D. thesis, TU Dresden, Germany, 2019.

(8) Pipek, J.; Mezey, P. G. A fast intrinsic localization procedure applicable for *ab initio* and semiempirical linear combination of atomic orbital wave functions. *The Journal of Chemical Physics* **1989**, *90*, 4916.

(9) Berning, A.; Schweizer, M.; Werner, H.-J.; Knowles, P. J.; Palmieri, P. Spin-orbit matrix elements for internally contracted multireference configuration interaction wavefunctions. *Molecular Physics* **2000**, *98*, 1823.

(10) Mitrushchenkov, A.; Werner, H.-J. Calculation of transition moments between internally contracted MRCI wave functions with non-orthogonal orbitals. *Molecular Physics* **2007**, *105*, 1239.

(11) Shao, Y. C.; Karki, B.; Huang, W.; Feng, X.; Sumanasekera, G.; Guo, J.-H.; Chuang, Y.-D.; Freelon, B. Spectroscopic Determination of Key Energy Scales for the Base Hamiltonian of Chromium Trihalides. *The Journal of Physical Chemistry Letters* **2021**, *12*, 724.

(12) van Veenendaal, M. *Theory of Inelastic Scattering and Absorption of X-Rays*; Cambridge: Cambridge University Press, 2015.

(13) Als-Nielsen, J.; McMorrow, D. *Elements of Modern X-ray Physics*; Wiley, 2011.

(14) Bogdanov, N. A.; Bisogni, V.; Kraus, R.; Monney, C.; Zhou, K.; Schmitt, T.; Geck, J.; Mitrushchenkov, A. O.; Stoll, H.; van den Brink, J. et al. Orbital breathing effects in the computation of x-ray *d*-ion spectra in solids by *ab initio* wave-function-based methods. *Journal of Physics: Condensed Matter* **2017**, *29*, 035502.

(15) Wilson, A. K.; Woon, D. E.; Peterson, K. A.; Dunning, T. H. Gaussian basis sets for use in correlated molecular calculations. IX. The atoms gallium through krypton. *The Journal of Chemical Physics* **1999**, *110*, 7667.
